# HIV infection in severely malnourished children in Kumasi, Ghana: a cross-sectional prospective study

**DOI:** 10.1186/1471-2431-13-181

**Published:** 2013-11-09

**Authors:** Serwah Bonsu Asafo-Agyei, Sampson Antwi, Samuel Blay Nguah

**Affiliations:** 1 Department of Child Health, Komfo Anokye Teaching Hospital, Kumasi, Ghana; 2 School of Medical Sciences, Kwame Nkrumah University of Science and Technology, Kumasi, Ghana

**Keywords:** Malnutrition, HIV, AIDS, SAM, PITC

## Abstract

**Background:**

The Human Immunodeficiency Virus (HIV) epidemic has adversely affected the nutritional status and mortality of children in Africa. This study assessed the disease burden, predictive clinical features and outcomes for children with severe acute malnutrition (SAM) and concomitant HIV infection.

**Methods:**

This was a cross-sectional prospective study of children with SAM aged between 3 months and 13 years admitted to the nutritional rehabilitation unit (NRU) of Komfo Anokye Teaching Hospital. Socio-demographic, anthropometric and clinical data were documented and HIV serostatus established with 2 rapid HIV antibody tests and Enzyme-linked immunosorbent assay, if indicated. HIV viral polymerase chain reaction testing was not available at the time of the study. Logistic regression analyses were used to identify significant clinical predictors of HIV seropositivity.

**Results:**

Sixty-seven (27.2%, 95% CI = 21.8-33.3%) of the 246 study children had positive HIV antibody results. Uptake of provider-initiated HIV testing and counselling was 100%. Amongst children aged 18 months and over, the HIV seroprevalence was 28.3% (30/106). HIV seropositivity was strongly associated with prolonged fever, cough and diarrhoea; oral thrush, generalised lymphadenopathy and pulmonary tuberculosis (p value < 0.001 for all parameters). The presence of ≥ 3 of the first 5 aforementioned parameters was highly specific (98.3-100%) for HIV seropositivity in study children. HIV seropositivity was also significantly associated with cough, vomiting, lethargy/altered consciousness, skin rash and hepatomegaly (p value < 0.05 for all parameters). Overall mortality rate was 17.5%, with HIV seropositive children having a significantly higher mortality rate (37.8% versus 10.1%; p value < 0.001) and a lower rate of weight gain (2.4 g/kg/day versus 7.0 g/kg/day; p value < 0.001).

**Conclusions:**

HIV testing was well accepted by parents/carers and should be offered in all NRUs. There was a high HIV seroprevalence among children with SAM and a significantly poorer outcome in mortality and weight gain. Some clinical features were identified to be predictive of HIV seropositivity and could be useful as indicators to prompt further investigation and/or referral in resource limited settings where HIV test kits are unavailable. This would aid in the early detection and comprehensive management of the HIV seropositive child with SAM.

## Background

The Human Immunodeficiency Virus (HIV) pandemic continues to plague many countries in the developing world [1]. At the end of 2010, 3.4 million children under 15 years were estimated to be living with the virus globally [1]. More than 90% of these were in sub-Saharan Africa [1]. Ghana continues to have a generalised HIV epidemic, with a national HIV prevalence of 1.5% in 2011 [2]. In the same year, an estimated 30,401 children under 15 years were living with HIV/AIDS [2].

Malnutrition, another condition prevalent in developing countries, contributes to more than a third of under-five deaths globally [3]. Malnutrition is endemic in many parts of sub-Saharan Africa. In an anthropometric assessment of children aged > 3 months to < 5 years attending the outpatient clinic of Komfo Anokye Teaching Hospital (KATH), Antwi [4] found 251/1182 (21.2%) to be wasted, with 48/1182 (4.1%) being severe.

HIV and malnutrition are intricately interwoven. Clinically, unexplained severe wasting or severe malnutrition not responding to standard therapy is classified as Stage IV in paediatric AIDS [5]. Both severe malnutrition and HIV have a deleterious effect on the immune system [6] and their clinical presentations overlap with many similarities. In resource limited settings (RLS) where laboratory diagnosis of HIV is not always possible, it would be useful to have a clinical algorithm that will raise a clinical suspicion of HIV infection in children with SAM. While some studies [7-9] suggest that certain clinical features and co-morbidities may be more predictive of HIV infection in SAM, this has not been consistently demonstrated in published literature [10]. Clinical features reported as being predictive of HIV infection in children with SAM include lymphadenopathy, oral candidiasis [7,9,11], skin disorders, hepatomegaly [7,11], persistent diarrhoea [8], chronic discharging ears [9] and prolonged fever [7]. Clinical parameters that aid in the presumptive diagnosis of HIV in children have been summarised in the Integrated Management of Childhood illness (IMCI) algorithm for suspected symptomatic HIV infection [12].

Studies suggest that between 8.6-54.0% of all children admitted to inpatient nutrition rehabilitation units (NRUs) in sub-Saharan Africa are HIV infected [8-11,13,14]. No such comparable published data exist for Ghana. Current World Health Organisation (WHO) guidelines (2012) recommend provider-initiated HIV testing and counselling (PITC) in clinical settings for all children in countries with an HIV prevalence of ≥ 1% in the general population [15]. PITC has been shown to be acceptable and effective in these settings [16]. Despite these recommendations, Baggaley et al. [17] found that only 9.5% of inpatients (adults or children) were offered PITC by countries that have adopted the WHO PITC policy. Inadequate testing for HIV infection is the major reason why less than one quarter of children eligible for treatment are accessing antiretroviral therapy [1]. Early identification of HIV infection will aid in reducing morbidity and mortality in both the children and their families.

Case fatality rates reported in various NRUs in the African sub-region have been unacceptably high [9,10,13]. The Sphere international standard [18] of less than 10% for therapeutic care has remained elusive for many of these NRUs. This has been attributed by some authors to the high prevalence of HIV infection and difficulties at the time of the studies in accessing reliable HIV testing and anti-retroviral drugs (ARVS) [19,20].

This study was therefore conducted to determine the uptake of PITC and the prevalence of HIV seropositivity among children with SAM admitted to the NRU of KATH. It also assessed the clinical predictors and outcome of HIV seropositivity in the study population.

## Methods

A cross-sectional, prospective study of children aged between 3 months and 13 years with SAM admitted to the NRU of KATH was carried out between February 2010 and October 2010. Ethical approval was obtained from the Committee on Human Research, Publications and Ethics of KATH/Kwame Nkrumah University of Science and Technology, Kumasi, Ghana.

KATH is a tertiary teaching hospital situated in Kumasi, the second largest city in Ghana. It is a referral centre for the Kumasi metropolis and the whole northern sector of Ghana. It has a paediatric NRU established in 2004 that admits children with SAM aged between 3 months and 13 years. All children admitted to the NRU with SAM, defined in this study as weight-for-height z-score (WHZ) < -3 SD of the median WHO reference values [21] or with symmetrical oedema attributable to malnutrition [22], were invited to participate. The only exclusion criterion was refusal of parental consent. Six parents refused to give consent and were excluded from the study. However, their children were offered PITC as per the guidelines of the unit. An estimated sample size of 246 was calculated based on a prevalence of 17.4% reported by Fergusson et al. [23] and a response rate of 90%. Recruitment was done prospectively until the sample size was reached.

The recruitment process was started within the first 24 hours of admission, after the attending medical team had completed their initial assessment and managed life threatening complications. For all eligible children, the study was explained to the parent or guardian, and written informed consent was sought. The child was then enrolled if the parent/guardian consented.

PITC was offered to all children admitted with SAM as per the management protocol of the NRU. Parents/carers of patients were referred to the Voluntary Counselling and Testing (VCT) centre to undergo pre- and post-test counselling. At the VCT centre, the child was tested for HIV using blood sample from a finger prick after the pre-test counselling. For those children who tested positive, their parents were also offered HIV testing. Testing for HIV was done by trained counsellors at the counselling unit of the hospital and consisted of 2 rapid HIV antibody tests (First response and OraQuick). Discordant results were retested with ELISA. PCR was not available for testing in children aged < 18 months at the time of the study. These children were deemed as HIV exposed (not necessarily HIV infected) when they tested positive with HIV antibody test. Parents or caretakers were interviewed and study patients examined. Four trained nutritionists assisted with the anthropometric measurements. Relevant laboratory, socio-demographic, anthropometric and clinical data were then recorded onto a case report form. Data was collected specifically for the purposes of this study throughout admission until discharge by the principal investigator. A diagnosis of urinary tract infection (UTI) was made only if it was culture proven. Severe anaemia was diagnosed if the haemoglobin level was less than 5 g/dl. Dehydration was assumed if the patient had frequent watery diarrhoea or vomiting. A diagnosis of pulmonary tuberculosis (PTB) was made based on a combination of the clinical presentation, a history of contact with PTB or chronic cough and results from laboratory investigations (mainly chest radiograph and Ziehl Neelsen stain of gastric lavage/sputum). A diagnosis of bacteraemia/septicaemia was made if there was a positive blood culture isolate. Malaria was diagnosed only if malaria parasites were seen on blood film.

Patients were nutritionally rehabilitated with F-75/F-100 and co-morbidities were treated. HIV seropositive children were referred to the KATH paediatric HIV clinic for comprehensive management and follow-up. A confirmatory HIV antibody test was done at 18 months in HIV exposed children and the results were documented.

On completion of data entry, entered data was transported to Stata Intercool version 11.2 for analysis. Any variable with significant missing data was excluded from the analysis. Categorical variables such as the sex and some clinical presentations were analysed and presented as percentages with their 95% confidence intervals. Continuous variables were however analysed and presented as their means with standard deviation for normally distributed variables, and median with inter-quartile ranges for skewed variables. Associations between the various clinical presentation and HIV status were done and reported as risk ratios (RR) with their corresponding 95% confidence intervals and p-values using Fisher’s exact test. A stepwise logistic regression (Wald chi-square statistic) was used to identify the most significant independent clinical predictors of HIV seropositivity. The diagnostic parameters (sensitivities, specificities, negative and positive predictive values) of the identified clinical predictors of HIV seropositivity were then determined. The clinical predictors were then combined in an algorithm to determine the likelihood of having HIV seropositivity based on the number of predictive clinical features a child presents with. For all analysis a two sided p-value of less than 0.05 was considered statistically significant.

## Results

### HIV seropositivity and socio-demographics characteristics

A total of 246 children were recruited for the study. One hundred and eleven (45.1%) were males. The median age was 15 months (IQR: 10–24 months). One hundred and forty children were aged < 18 months. Only 6 children were aged more than 5 years. The oldest child was aged 10 years. Fifteen patients (6.1%) were orphans; of these, 10 (4.1%) were maternal orphans, 4 (1.6%) were paternal orphans and 1 (0.4%) had lost both parents. PITC was done for all the patients giving a test uptake of 100%. Sixty-seven (27.2%, 95% CI = 21.8-33.3%) of the 246 children had positive HIV antibody test results. Among the 106 children aged 18 months or older in whom HIV antibody test was confirmatory; 30 tested HIV seropositive (28.3%, 95% CI = 20.0 - 37.9%). Of the remaining 140 children, 37 (26.4%) tested HIV seropositive (HIV exposed). The mean age of HIV seropositive study subjects was 26 months whereas that of HIV seronegative patients was 17.7 months (p-value = 0.013). All children aged more than 5 years were HIV seropositive. Five of these presented with severe wasting and one had oedematous malnutrition. Ten of the 15 orphans were HIV seropositive whereas 5 were HIV seronegative.

### HIV seropositivity, anthropometric indices and clinical features

The mean WHZ for wasted children ≤ 5 years (195 children) was -4.6 (SD =1.0). HIV seropositive patients were significantly more wasted; with lower mean weight for height z-scores. (-5.1 in comparison to -4.4 for seronegative children). Two hundred patients (81.3%) had severe wasting whereas 46 patients (18.7%) had oedematous malnutrition. Eighteen (39.1%) of the oedematous children had severe wasting (marasmic-kwashiorkor). Amongst HIV seropositive children, 7/67 (10.4%) had oedematous malnutrition. HIV seropositive patients were significantly more likely than HIV seronegative patients to have severe wasting than oedematous malnutrition (89.6% of HIV seropositive children had severe wasting as against 78.2% in HIV seronegative children; p value = 0.042).

The association between HIV seropositivity, clinical features and co-morbidities is shown in Tables 1 and 2 respectively*.* HIV seropositivity was strongly associated with prolonged fever, cough and diarrhoea; oral thrush, generalised lymphadenopathy and pulmonary tuberculosis (p value < 0.001 for all parameters). HIV seropositivity was also significantly associated with cough, vomiting, lethargy or altered consciousness, skin rash, hepatomegaly and bacteraemia (p value < 0.05 for all parameters). Severe anaemia was however more likely in HIV seronegative patients.

**Table 1:** Association of clinical features with HIV seropositivity (both exposed and infected)

	**HIV status n (%)**			
**Clinical variable**	**Negative**	**Positive**	**RR (95% CI)**	**P-value**
	**n-179**	**n-67**		
Fever	142 (79.3)	56 (83.6)	1.1 (0.9 - 1.2)	0.454
*Prolonged fever	14 (7.8)	**31 (46.3)**	5.9 (3.4 - 10.4)	**< 0.001**
Diarrhoea	80 (44.7)	33 (49.3)	1.1 (0.8 - 1.5)	0.523
*Prolonged diarrhoea	10 (5.5)	16 (23.9)	4.3 (2.0 - 8.9)	**< 0.001**
Cough	64 (35.8)	39 (58.2)	1.6 (1.2 - 2.2)	**< 0.001**
*Chronic cough	8 (4.5)	27 (40.3)	9.0 (4.3 - 18.8)	**< 0.001**
Poor Feeding	134 (74.9)	57 (85.1)	1.1 (1.0 - 1.3)	0.087
Lethargy/altered consciousness	85 (47.5)	46 (68.7)	1.4 (1.2 - 1.8)	**0.003**
Vomiting	76 (42.5)	39 (58.2)	1.4 (1.1 - 1.8)	**0.028**
Fast/difficult breathing	41 (22.9)	17 (25.4)	1.1 (0.7 - 1.8)	0.685
Pallor	82 (45.8)	24 (35.8)	0.8 (0.5 - 1.1)	0.159
Hepatomegaly	56 (31.3)	32 (47.8)	1.5 (1.1 - 2.1)	**0.016**
Oral thrush	32 (17.9)	46 (68.7)	3.8 (2.7 - 5.5)	**< 0.001**
Skin rash	20 (11.2)	17 (25.4)	2.3 (1.3 - 4.1)	**0.006**
Generalised lymphadenopathy	3 (1.7)	30 (44.8)	26.7 (8.4 - 84.6)	**< 0.001**
Splenomegaly	28 (15.6)	15 (22.4)	1.4 (0.8 - 2.5)	0.215

*Duration lasting 14 days or longer.

n = number.

RR = Relative risk.

**Table 2:** Association of HIV with other severe acute malnutrition (SAM) complications and co-morbidities in the study population

	**HIV status n (%)**			
**Clinical variable**	**Negative**	**Positive**	**RR (95% CI)**	**P-value**
	**n-179**	**n-67**		
Bacteraemia	55 (31.1)	30 (44.8)	1.5 (1.0 - 2.1)	**0.045**
UTI	32 (17.9)	16 (23.9)	1.3 (0.8 - 2.3)	0.290
*Dehydration	34 (19)	11 (16.4)	0.9 (0.5 - 1.6)	0.642
SA	35 (19.6)	5 (7.5)	0.4 (0.2 - 0.9)	**0.021**
Malaria	28 (15.6)	6 (9)	0.6 (0.2 - 1.3)	0.176
Pneumonia	23 (12.8)	10 (14.9)	1.2 (0.6 - 2.3)	0.671
PTB	5 (2.8)	18 (26.9)	9.6 (3.7 - 24.9)	**< 0.001**

UTI = Urinary tract infection.

PTB = Pulmonary tuberculosis.

SA = Severe anaemia.

n = number.

RR = Relative risk.

*Dehydration was usually associated with diarrhoea and/or vomiting.

### Development of clinical algorithm

Table 3 shows the sensitivities and specificities of the strongest clinical predictors of HIV as independent variables and when grouped together as a clinical algorithm. The presence of 3 or more symptoms was highly specific for HIV seropositivity in study children, and could be utilised for presumptive diagnosis of HIV infection in children with SAM.

**Table 3:** Predictors of HIV seropositivity in study population and their sensitivity and specificity

**Clinical variable**	**Sensitivity% (95% CI)**	**Specificity% (95% CI)**	**PPV% (95% CI)**	**NPV% (95% CI)**
Prolonged diarrhoea	23.9 (14.3 - 35.9)	94.4 ( 90.0-97.3)	61.5 (40.6 - 79.8)	76.8 (70.7 - 82.2)
Chronic cough	40.3 (28.5 - 53.0)	95.5 (91.4 - 98.1)	77.1 (59.9 - 89.6)	81.0 (75.1 - 86.1)
Prolonged fever	46.3 (34.0 - 58.9)	92.2 (87.2 - 95.7)	68.9 (53.4 - 81.8)	82.1 (76.1 - 87.1)
Oral thrush	68.7 (56.2-79.4)	82.1 (75.7-87.4)	59.0 (47.3-70.0)	87.5 (81.5-92.1)
Generalised lymphadenopathy	44.8 (32.6-57.4)	98.3 (95.2-99.7)	90.9 (75.7-98.1)	82.6 (76.9-87.5)
Sensitivities and specificities of a clinical algorithm consisting of above variables				
No of parameters identified	Sensitivity%	Specificity%	Correctly Classified%	
≥ 1	92.5	72.1	77.6	
≥ 2	68.7	92.2	85.8	
≥ 3	41.7	98.3	82.9	
≥ 4	16.4	100.0	77.2	
5	4.8	100.0	74.0	

PPV - Positive predictive value.

NPV - Negative predictive value.

### Nutritional recovery and mortality

HIV seropositive children had a significantly (p < 0.001) lower mean weight gain per kilogram per day (2.4 g/kg/day, 95% CI = 0.7-4.1 g/kg/day) than HIV seronegative subjects (7.0 g/kg/day, 95% CI = 5.8-8.2 g/kg/day). Two hundred and three (82.5%) patients survived and 43 (17.5%) patients died during admission. HIV seropositive children had a significantly higher risk of dying (mortality rates in HIV seropositive 37.8% (25/67) versus 10.1% (18/179) in HIV seronegative; p value < 0.001). Eighteen (48.6%) out of the 37 HIV exposed children died during admission, 1 (2.7%) died at home after discharge, 12 (32.4%) tested HIV seropositive at 18 months and 6 (16.2%) were lost to follow-up.

## Discussion

### Prevalence and impact of HIV

This study found a PITC uptake of 100% which could be attributed to the '*opt out*’ approach utilised and the skills of the trained counsellors at KATH. Mutanga et al. [16] also reported a comparable PITC uptake of 98.2% in Zambia.

The HIV seroprevalence of 27.2% in this study is comparable to the 29.2% overall prevalence reported by Fergusson et al. [20] in a meta-analysis of seventeen African studies. Among HIV seronegative children with SAM, the mortality rate almost met the minimum Sphere international standard [18] of less than 10%. In contrast, the mortality rate in HIV seropositive children was almost 4 fold higher. Other studies have similarly reported a higher mortality rate in HIV seropositive compared to HIV seronegative children with SAM [10,19,20,23]. The higher mortality rate and the lower rate of weight gain in HIV seropositive children could be due to the higher prevalence of potentially life-threatening co-morbidities like PTB, bacteraemia and diarrhoeal diseases. It could also be related to the fact that HIV-infected children are more likely to have complicated case management issues like multiple pathology, drug-drug interactions and drug toxicities. Excler et al. [24] found that HIV seropositive children with SAM did not respond well to high protein-energy diet whilst Fergusson et al. [23] found no significant difference in rate of weight gain by HIV status, though the latter found a higher mortality in the HIV seropositive group. Thus, in view of the poorer prognosis in HIV seropositive children with SAM, there should be well developed linkages between NRUs and HIV clinics. Where possible, health personnel working in NRUs should be trained in the management of paediatric HIV in a manner similar to the existing linkage between TB and HIV clinics. In this study, majority of the NRU staff were also part of the HIV and TB clinical teams.

### Anthropometric and clinical variables

HIV seropositive children were more likely to have severe wasting than oedematous malnutrition, a finding consistent with reports from other authors [7-10]. Children recruited in this study consisted of both those with primary malnutrition and those with malnutrition secondary to HIV and other chronic medical conditions like cerebral palsy, PTB and congenital heart disease. Traditionally, primary SAM has been reported to occur in children less than 5 years [25]. Relatively older children are likely to have underlying organic disease or infection contributing to their malnutrition. Children > 5 years with SAM should therefore be thoroughly assessed for secondary causes of malnutrition.

Both severe malnutrition and HIV cause immunosuppression, predisposing children to opportunistic infections (OIs) with its attendant clinical features reported in this study. Consistent with findings from this study, Prazuck et al. [11] in Burkina Faso also found a strong association of HIV infection with oral thrush (p < 0.0006) and lymphadenopathy (p < 0.0001). Yeung et al. [26], however, found no significant differences in clinical features in rural South African children with and without HIV. However, the study included children who were well nourished or moderately malnourished. Bacteraemia and PTB were significantly associated with HIV seropositivity in this study. The clinical experience of HIV/TB co-infection in childhood has also been well documented by other authors [27-29]. However, some authors found no significant association between HIV and tuberculosis in children with SAM [10]. Severe anaemia was significantly commoner in HIV negative participants. The reason for this was not clear. It may be due to the relatively higher incidence of malaria in the HIV seronegative patients recorded in this study. Studies on HIV and malaria interactions in children have been inconclusive [30]. Osterbauer et al. [31] found that HIV-unexposed infants had a higher risk of malaria parasitaemia compared to HIV-exposed infants (p = 0.004), although there was no significant association between HIV-exposure status and malaria parasitaemia after controlling for the use of malaria preventative measures. In contrast, Chinkhumba et al. [32] reported that HIV-infected children were more likely to have lower haemoglobin levels.

### Development of a clinical algorithm

Survival has improved dramatically however challenges remain. In a country like Ghana with a relatively low HIV prevalence, health workers may not have a high enough index of suspicion of HIV infection to request PITC under appropriate settings. The child’s clinical symptomatology may be wrongly attributed to the SAM. HIV seropositive children may die before they are diagnosed or before they can access ARVs. Inarguably, a clinical algorithm for presumptive diagnosis of HIV has its limitations. Nevertheless, it could be useful in RLS where facilities for testing may be lacking or are in short supply. It could aid in prompt referral for VCT and comprehensive management. The utility of such clinical algorithms have been recognised and their use encouraged in the absence of appropriate laboratory tests [33].

## Conclusions

HIV seroprevalence in severely malnourished Ghanaian children is high (27.2%). There was excellent uptake of PITC (100%). Thus, NRUs could serve as important entry points for early detection and comprehensive management of HIV infected children and their families.

Overall, our findings support well recognised clinical features of symptomatic HIV infection in children. In the absence of laboratory facilities, a strong suspicion of HIV seropositivity should be entertained in a child with SAM who has 3 or more of the following clinical features; prolonged fever, cough and diarrhoea; oral thrush and generalised lymphadenopathy. PTB in a severely malnourished child is also strongly suggestive of HIV seropositivity.

HIV seropositive patients had higher mortality rates and lower rate of weight gain. HIV seropositive children should thus be screened rigorously for OIs and monitored closely during rehabilitation.

## Limitations of the study

Viral antigen testing was not available at the time of the study for children <18 months of age who tested positive with HIV antibody test. These children were followed up for confirmatory HIV test at 18 months but 19/37 (51.4%) died before 18 months and 6 (16.2%) were lost to follow-up.

It was not determined whether the cause of the SAM was primary, although detected secondary causes of malnutrition were documented.

Also, information on certain aspects of the history like contact with pulmonary tuberculosis was lacking in orphaned or abandoned children. However, this was an infrequent occurrence.

Although the study period fairly covered the 2 major seasons in Ghana, it may not be truly representative of children admitted to the NRU throughout the year.

## Abbreviations

ARVS: Antiretroviral drugs; ELISA: Enzyme-linked immunosorbent assay; HIV: Human immunodeficiency virus; KATH: Komfo Anokye teaching hospital; NRU: Nutritional rehabilitation unit; OIs: Opportunistic infections; PCR: Polymerase chain reaction; PITC: Provider-initiated HIV testing and counselling; PTB: Pulmonary tuberculosis; RLS: Resource limited settings; SAM: Severe acute malnutrition; UTI: Urinary tract infection; VCT: Voluntary counselling and testing; WHO: World Health Organisation; WHZ: Weight-for-height Z-score.

## Competing interests

The authors declare that they have no competing interests.

## Authors’ contributions

SBAA sought for ethical clearance, participated in the study design, data collection and entry and manuscript writing. SA and SBN participated in study design, supervision and completion of final manuscript, and SBN additionally performed the statistical analysis. All authors read and approved the final manuscript.

## Pre-publication history

The pre-publication history for this paper can be accessed here:

http://www.biomedcentral.com/1471-2431/13/181/prepub
